# Today and tomorrow: In memoriam Vesna Andrijević-Matovac (1960-2011)

**DOI:** 10.3325/cmj.2012.53.83

**Published:** 2012-02

**Authors:** Nika Matovac

Dear readers,

In two and almost a half years of writing this column my mum, Vesna Andrijević-Matovac, and I, Nika Matovac, shared with you a true and very intimate story of a woman with breast cancer and her daughter. In these columns, you have witnessed our great moments, sad moments, and devastating moments, you have been a part of our life. Now, as everything has its beginning and its end, it is time to say goodbye, to close this column. But don’t ever forget that this is just a beginning, just a way to provide directions what to do in such a situation and how to help.

## Our future

Future is what all patients with breast cancer are dreaming of, future for them seems like something strange, like something they should not think of, but living with my mum changed my childish opinion (well, I was only 11 years old when my mum got her diagnosis) that every person with cancer is going to die, die soon… I may say that my mum was a dreamer, but at the end she started living her dream. Her dream was to establish an association that would help other patients with cancer, to be there for everything they might need. “EVERYTHING for HER!” was established on March 21, 2008. The dream started and my mum’s wish to help others became reality. At first, the office of “EVERYTHING for HER!” was a room in our apartment and then the second wish was born. “I want a place where people with cancer can come, can talk to a psychologist, can get a support and feel like at home!”, my mum would say, and believe it or not, in May 2010 she started living her second dream – the Center for Psychological Assistance for women with cancer “EVERYTHING for HER!” was opened in Zagreb, Kneza Mislava 10 (*http://www.svezanju.hr/*). All I wanted to show is that a dream can give you strength to live and work and one day to live this dream. What I am very proud of is that my mum’s dream lives on and that it will help others to live their dreams and not to be afraid of dreaming.

Tomorrow is something most of us don’t even think of as a gift, we consider it normal, but those who are fighting for health, those with any disease, find it to be a miracle, they find it to be a new opportunity, but also it opens for them, again and again, the chamber of mixed feelings, happiness, will for cure, sadness, and fear, fear of the future.

In the last 10 years, I have learned that it is not easy to be a patient for a number of reasons, but now there is help. As a future doctor I may say that doctors, although they may not think so, can do a lot about how patient will deal with his or her illness, only if they show some humanity. Doctors should not be afraid to show some feelings and empathy. I know that probably the most stressful thing in a doctor’s career is to give a wrong diagnosis, to say a devastating truth, to give bad news. We have to learn how to do this and stay strong, but also how not to hide from somebody’s reality behind a marble face. Center for Psychological Assistance for women with cancer “EVERYTHING for HER!” will help patients to deal with their fear of tomorrow, give them strength to dream and to live their dreams, and it will be patients’ second home. Though society is something we cannot change very fast, as individuals we can do a lot, and by thinking this way our society will change faster and listen more to patients’ wishes, needs, and dreams.

## February 14, 2012 (a year after mum’s death)

A year has passed since I got the news that my mum is not with us anymore, and now when somebody asks me how I feel and whether I miss her, I don’t know how to react, I don’t know why such questions are even asked. I think that everybody misses and will always miss their family member or friend, especially parents. I miss my mum very much, but I am also very proud that I had a mum with such strength and wish to help others despite her illness. I won’t be modest and I will say that my mum’s dream has changed and will change many lives, and that’s why I think that my mum still lives through the association “EVERYTHING for HER!”, which gives me strength to move on and not to escape from my family, and my family are all people, regardless of the culture, color, and language. We are all one big family, we are all here to open our soul, to look with our heart, to love, and to be loved.

